# 

**Published:** 1880-03-20

**Authors:** 


### TABLE OF CONTENTS.

ORIGINAL AND SELECTED ARTICLES.

Reply to Prof. Joynes' Paper on "Quarantine by the General Government," and to Prof. Chaille on "Castration and Spaying as a Means of Arresting Syphilis," by Dr. Alban S. Payne, Professor of Practice, in Southern Medical College, continued from Feb. Number, 81. Sequellæ of Rheumatism, by A. G. Hobbs, M.D., 86. Impacted Fracture of the Femoral Neck, by A. F. Kinne, A.M., M.D., 90. Topical Uses of Ergot, 91. Post-Mortem of a Case of Hodgkin's Disease, 93. Malignant Remittent Fever, 95. Signs of Death by Drowning; Duboisia and Atropia, 97.

ABSTRACTS AND GLEANINGS.

Nasal Catarrh, 98. Recent Decisions, 100. Salicin and Salicylic Acid in Acute Rheumatism, 101. Tracheotomy in Croup, 103. Cough from elongated Uvula—Operation, 104. Forgetting Personal Identity, 105. Intra-Uterine Medication; Hiccough, 107. New Treatment of Placenta Prævia by Ferri Persulphatis; Pulsatilla for Dysmenorrhœa; Remedies for Hypodermic Use; Failure of the Audiphone, 108. Milk and Lime-Water; Poisons and Antidotes; Operation for Pterygium; Eserine, 109. Hyoscyamia in Insanity; Hot Water for the Induction of Premature Labor; Aspiration of the Bladder for Retention of Urine, 110.

SCIENTIFIC ITEMS.

About Snakes; Voice in Fishes, 111. Submarine Telephoning; Novel Theory about Diamonds; The King of Ploughs, 112.

PRACTICAL NOTES AND FORMULÆ.

Bedford Iron and Alum Mass; Parvules; Treatment of Obstinate Eczema, 113. Glycerine in Coughs, 114. Remedy for Corns; To Relieve Itching: For Asthma, Croup, etc.; Mixture for Acute Gonorrhœa; For Night Sweats, 115. Quinine in Whooping Cough; Recent Suggestion for Ozena; Nervous Debility, Dyspepsia, etc.

EDITORIAL AND MISCELLANEOUS.

Editorial Notices; Medical Associations; Convention on Vital Statistics, 117. Southern Medical College Commencement, 118. Book Notices, 119. Special Notices, 120.

